# Effects of post-capture ventilation assistance and elevated water temperature on sockeye salmon in a simulated capture-and-release experiment

**DOI:** 10.1093/conphys/cot015

**Published:** 2013-06-11

**Authors:** Kendra A. Robinson, Scott G. Hinch, Marika K. Gale, Timothy D. Clark, Samantha M. Wilson, Michael R. Donaldson, Anthony P. Farrell, Steven J. Cooke, David A. Patterson

**Affiliations:** 1 Pacific Salmon Ecology and Conservation Laboratory, Department of Forest and Conservation Sciences, University of British Columbia, Vancouver, BC, Canada V6T 1Z4; 2 Australian Institute of Marine Science, PMB 3, Townsville MC, Queensland 4810, Australia; 3 Fish Ecology and Conservation Physiology Laboratory, Department of Biology and Institute of Environmental Science, Carleton University, Ottawa, ON, Canada K1S 5B6; 4 Department of Zoology and Faculty of Land and Food Systems, University of British Columbia, Vancouver, BC, Canada V6T 1Z4; 5 Fisheries and Oceans Canada, Science Branch, Cooperative Resource Management Institute, School of Resource and Environmental Management, Simon Fraser University, Burnaby, BC, Canada V5A 1S6

**Keywords:** Exhaustive exercise, facilitated recovery, fisheries, revival, stress, survival

## Abstract

The live release of wild adult Pacific salmon (*Oncorhynchus* spp.) following capture is a management tactic often used in commercial, aboriginal, and recreational fisheries. Fisheries capture and handling can be both exhausting and stressful to fish, which can limit their ability to swim and survive after release. As a result, researchers have assessed methods intended to improve post-release survival by assisting the flow of water over the gills of fish prior to release. Such approaches use recovery bags or boxes that direct water over the gills of restrained fish. This study evaluated a method of assisting ventilation that mimics one often employed by recreational anglers (i.e. holding fish facing into a current). Under laboratory conditions, wild Fraser River sockeye salmon (*Oncorhynchus nerka*) either received manual ventilation assistance for 1 min using a jet of water focused at the mouth or were left to recover unassisted following a capture-and-release simulation. A control group consisted of fish that were not exposed to the simulation or ventilation assistance. The experiment was conducted at 16 and 21°C, average and peak summer water temperatures for the Fraser River, and fish survival was monitored for 33 days. At 21°C, all fish perished within 3 days after treatment in all experimental groups, highlighting the consequences of handling adult sockeye salmon during elevated migration temperatures. Survival was higher at 16°C, with fish surviving on average 15–20 days after treatment. At 16°C, the capture-and-release simulation and ventilation assistance did not affect the survival of males; however, female survival was poor after the ventilation assistance compared with the unassisted and control groups. Our results suggest that the method of ventilation assistance tested in this study may not enhance the post-release survival of adult Fraser River sockeye salmon migrating in fresh water.

## Abstract (precis)

The authors evaluated a method of assisting the ventilation of adult sockeye salmon in an attempt to enhance post-release survival after fisheries capture at moderate and peak water temperatures. Though comparable recovery methods are often used by recreational anglers, the authors found this to be ineffective in enhancing post-release survival.

## Introduction

Understanding the physiological demands placed on a fish during fisheries capture through to recovery (assuming it is to be released) are key to evaluating conservation-driven attempts to aid recovery processes and reduce post-release mortality. Fisheries capture and handling results in a stress response and physiological exhaustion associated with both aerobic and anaerobic metabolic activity. The physiological stress response is initiated with the release of catecholamines and corticosteroids, which stimulate cardiorespiration to meet mounting tissue oxygen requirements (Pickering and Pottinger, 1995). Encounters with fisheries gear often trigger anaerobic responses, which tend to be faster and result in an oxygen deficit as tissue energy demands rapidly exceed that which can be produced aerobically (Pickering and Pottinger, 1995). Burst swimming, a short duration of rapid movement typically associated with gear encounters, uses the anaerobic breakdown of glycogen by white muscle fibres (Black *et al.*, 1966) to fuel attempts to evade or escape capture. The resulting lactate and metabolic protons accumulate in both muscle and blood, leading to altered acid–base status and osmoregulatory imbalance (Turner *et al.*, 1983; Wood, 1991; Milligan, 1996; Kieffer, 2000). Physiological recovery requires oxygen in excess of basal metabolic needs for processes such as high-energy phosphate replenishment, lactate clearance, and glycogen resynthesis (Wood, 1991). Thus, elevated cardiac and respiratory efforts continue well after the exhaustive burst swim events (Scarabello *et al.*, 1991; Wood, 1991; Thorarensen *et al.*, 1996; Lee *et al.*, 2003a, b). Although homeostasis can be regained, the stress of and recovery from fisheries capture-and-release events can place a considerable physiological load on fish that may directly affect their survival.

The duration of recovery needed for repeat burst events ranges from 40 min to several hours (reviewed by Wood, 1991; Milligan, 1996; Farrell *et al.*, 1998). In nature, such recovery durations make fish vulnerable to predators or fisheries recapture, or force fish to fall back downstream in fluvial systems. Factors surrounding capture can prolong the recovery process (Kieffer, 2000; reviewed by Cooke and Suski, 2005; Suski *et al.*, 2006). For example, air exposure (e.g. removal from net, unhooking, or photography) starves tissues of oxygen (Ferguson and Tufts, 1992), much like the effects of hypoxia, during the critical recovery period. Moreover, warm water during capture-and-release events can limit the maximal aerobic scope available and delay recovery times (Farrell *et al.*, 2008; Pörtner and Farrell, 2008; Eliason *et al.*, 2011). Consequently, the duration of recovery following a capture-and-release event can depend on the severity of stress associated with the particular capture event (Suski *et al.*, 2006) and the water temperature (Lee *et al.*, 2003a).

Adult sockeye salmon (*Oncorhynchus nerka*) that return to the Fraser River, BC, Canada during their once-in-a-lifetime spawning migration are captured in both marine and freshwater fisheries by commercial, aboriginal, and recreational fishers. In order to protect threatened sockeye salmon stocks and to ensure spawning escapement targets are met for all stocks, managers implement catch limits and temporal fishery closures. Fraser River recreational rod-and-reel anglers will often release sockeye salmon. In 2010, the recreational fishery released one-third (∼100 000) of sockeye salmon captured in the lower Fraser River (DFO, 2011) and 43% (∼63 000) of captured sockeye salmon the following year (DFO, 2012). However, little is known about the effects that capture-and-release events have on the survival of sockeye salmon. A recent telemetry experiment estimated that ∼35% of Fraser River sockeye salmon released from freshwater angling perished as a result of the capture-and-release event prior to reaching natal sub-watersheds (Donaldson *et al.*, 2011). In addition to capture-related stressors, Fraser River sockeye salmon experience warm summer river temperatures during their up-river migration. The mean summer temperature in the lower Fraser River has increased by ∼2°C from 1953 to 2006 (Patterson *et al.*, 2007), and there is concern that high-temperature events will increase in both frequency and duration in the future (Hague *et al.*, 2011). Indeed, high water temperatures have been correlated with in-river mortality during spawning migration (Macdonald *et al.*, 2010), and high temperatures at the time of capture and research tagging have also been associated with elevated post-release mortality in the wild (Martins *et al.*, 2011). Laboratory experiments conducted on Fraser River sockeye salmon found that fish which were exhaustively exercised in warm water and then exposed to air for 1 min had significantly depressed ventilation rates (Gale *et al.*, 2011) and were not likely to survive the following 24 h (Gale, 2011). These results suggest that releasing sockeye salmon which have not adequately recovered can leave fish vulnerable to delayed mortality, particularly in warm waters.

Various approaches to facilitating the post-capture recovery of adult Pacific salmon (*Oncorhynchus* spp.) have been evaluated. Most approaches involve ensuring adequate water flow across gill surfaces, thereby increasing the potential for more oxygen delivery during excess requirements (see Farrell *et al.*, 2001; Donaldson *et al.*, 2013; Nguyen *et al.*, 2013). For example, a device termed a Fraser Box is designed to force ventilation by restricting fish movement while orienting the fish into a water jet. This device promoted physiological recovery and reduced short-term (24 h) mortality for coho salmon (*Oncorhynchus kisutch*) that had been caught by a marine commercial gillnet (Farrell *et al.*, 2001). Other approaches that facilitate the flow of water over a fish's gills by manually moving the fish in an S-shape or back and forth in the water, or by orienting the fish upstream, are recommended to recreational anglers by North American natural resource agencies (Pelletier *et al.*, 2007); however, clear evidence of the efficacy of these manual recovery techniques is lacking (Arlinghaus *et al.*, 2007; Pelletier *et al.*, 2007).

The objective of this study was to evaluate manual ventilation assistance as a means of facilitating the recovery of adult migrating Fraser River sockeye salmon after a simulated fisheries capture-and-release event at a moderate and a high temperature. Capture and release were simulated by exhaustively exercising then air exposing fish and assessing how post-release survival was affected by exposing some of these fish to rapid water flow across their gills. Fish were held and oriented into a set water flow to simulate a simple technique that could easily be adopted and, in some cases, is already used by fishers. To confirm that the level of physiological disturbance the fish experienced was consistent with exhaustive exercise, blood samples were taken after treatment and analysed for metabolites. The temperature treatments reflected the average and peak temperatures encountered by river-migrating Fraser River sockeye salmon in order to examine the efficacy of this assisted ventilation technique over a broad thermal range.

## Materials and methods

### Study site and animals

Adult sockeye salmon were collected by beach seine on 10–13 August 2010 from the Fraser River at Chilliwack, BC, Canada soon after commencing their freshwater spawning migration. This capture locale is a popular area for recreational anglers targeting returning sockeye salmon. The river temperature during collection was 18–20°C. Fish were transported in aerated ∼14°C water using truck-mounted transport tanks to the Fisheries and Oceans Canada (DFO) Cultus Lake Salmon Research Laboratory (∼26 km), where passive integrated transponder tags (∼8.5 mm × 2 mm size, 134.2 kHz; Biomark Inc., Boise, ID, USA) were inserted into the coelomic cavity for individual identification. Fish were then transferred to 10 circular 1400 l aerated tanks (2 m diameter; 12–13 fish per tank) supplied with filtered and UV-sterilized circulating fresh water (LS-Permabead Filtration System; Integrated Aqua Systems Inc., Escondido, CA, USA) from Cultus Lake. The fish were held at 14°C for ≥15 h to recover from transport.

### Experimental design

A total of 103 fish (55 males and 48 females) were used in the experiment. On 14 August 2010, the temperature was gradually increased (by ∼0.5°C h^−1^) to 16 (five tanks) and 21°C (five tanks) to simulate the current average and record peak summer Fraser River temperatures experienced by migrating adult sockeye salmon (Patterson *et al.*, 2007). The experiment commenced ≥36 h after a constant temperature was reached.

Fish were subjected to one of three simulated fisheries capture-and-release treatments: (i) control [20 males (10 at 16°C and 10 at 21°C) and 13 females (eight at 16°C and five at 21°C)]; (ii) capture-and-release simulation without assisted ventilation [18 males (10 at 16°C and eight at 21°C) and 16 females (eight at 16°C and eight at 21°C)]; and (iii) capture-and-release simulation with assisted ventilation [17 males (10 at 16°C and seven at 21°C) and 19 females (nine at 16°C and 10 at 21°C)]. The simulated fisheries capture consisted of four experimenters leaning over an ∼800 l doughnut-shaped tank (2 m diameter; at the experimental temperature) to stimulate the fish to burst swim by touching the tails of the fish or vigorously splashing behind them. After 3 min of this manual chasing, fish were immediately dip netted and exposed to air for 1 min. Similar chasing techniques have been used to stimulate exhaustive exercise in fish (reviewed by Milligan, 1996; Kieffer, 2000) and have been refined for fisheries capture simulations (Gale *et al.*, 2011; Raby *et al.*, 2013). This simulation was not designed to impose physical injury, which could contribute to immediate and delayed mortality (reviewed by Chopin and Arimoto, 1995). The assisted ventilation technique involved orienting fish into a jet of water flow from a submersible pump for a maximum of 1 min. Opercular beats were observed as the experimenter held the fish with the mouth ∼20 cm from the jet outlet. The water speed, ∼0.50 m s^−1^ (as measured at the mouth), is similar to or greater than the water speed that migrating sockeye salmon are experiencing in the Fraser River and its tributaries (Hinch and Rand, 2000), and is similar to the water speed used in evaluating portable recovery bags for Fraser River sockeye salmon (∼0.1–0.4 m s^−1^; Donaldson *et al.*, 2013). If the fish became vigorous and attempted to escape, it was released (similar to an angler releasing an active fish), and the duration of manual ventilation assistance was recorded. Of the 36 fish subjected to the assisted ventilation technique, 12 were released prior to the completion of the 1-min treatment. Control fish were not subjected to the capture-and-release simulation or assisted ventilation technique.

After release, fish were rapidly transferred by dip net to individual rectangular holding tanks (∼100 l, 1 m × 0.5 m × 0.3 m deep) with fresh flowing water at the experimental temperature. Thirty minutes later, blood was sampled (see ‘Blood sampling and laboratory assays’ below) by transferring the fish by dip net to a flow-through, foam-lined trough. Fish were finally transferred to 7000 l aerated tanks (3 m diameter) at the appropriate experimental temperature for long-term monitoring. Control fish were dip netted directly from the 1400 l tanks and sampled for blood immediately before transfer to the 7000 l tanks. All fish were monitored for survival in their respective temperatures for up to 33 days, which represents the approximate time when these wild sockeye salmon would have arrived at their natal spawning grounds (Gilhousen, 1990).

Mortalities were examined to determine fork length (FL), body mass (*M*_B_) and sex. Opercular tissue (7 mm diameter) was sampled for DNA stock identification (Beacham *et al.*, 2005), which indicated that the fish were from Early Summer and Summer-run stock groups, a classification scheme used by fisheries managers to describe the timing of river entry. Early Summer and Summer-run sockeye salmon enter the river in July and August and experience overlapping river temperatures (Patterson *et al.*, 2007). These run-timing groups were pooled for analyses.

### Blood sampling and laboratory assays

A series of blood plasma variables were measured to assess the physiological response to the fisheries capture-and-release treatments and thereby allow for comparison with previous studies and ensure that our capture simulation accomplished the required exhaustion. Plasma cortisol can be measured as an indicator of the stress response (Wendelaar Bonga, 1997; Barton, 2002). Increases in plasma lactate and glucose metabolites are indicative of anaerobic activity and exercise stress, respectively (reviewed by Wood, 1991; Donaldson *et al.*, 2011). Haematocrit, haemoglobin, and mean corpuscular haemoglobin concentration (MCHC) can be used to assess changes associated with aerobic activity in the blood (Clark *et al.*, 2008; Donaldson *et al.*, 2010). Osmoregulatory variables [sodium (Na^+^), chloride (Cl^−^), potassium (K^+^), and osmolality] can be analysed to identify the acute response to exercise (reviewed by Milligan, 1996; Donaldson *et al.*, 2010, 2011) and chronic osmoregulatory conditions (Hruska *et al.*, 2010; Jeffries *et al.*, 2011).

Samples (∼3 ml) were obtained via caudal puncture using a heparinized vacutainer (detailed by Cooke *et al.*, 2005) and stored in a water–ice slurry for ≤1 h (Clark *et al.*, 2011). A hand-held haemoglobin analyser (HemoCue Hb 201^+^; HemoCue, Ängelholm, Sweden; calibrated for fish blood) was used on whole, well-mixed blood (Clark *et al.*, 2008). The haematocrit (expressed as a percentage) was quantified using haematocrit tubes centrifuged at 10 000 × *g* for 3 min. MCHC was calculated as follows: [haemoglobin]/([haematocrit]/100) (as by Donaldson *et al.*, 2010). The remaining whole blood was centrifuged at 7000 × *g* for 5 min. Plasma was isolated and flash frozen in 1.5 ml cryogenic vials in liquid nitrogen prior to storage at −80°C. Plasma was analysed for cortisol (Neogen enzyme-linked immunosorbent assay with Molecular Devices Spectramax 240pc plate reader), lactate and glucose (YSI 2300 Stat Plus analyser), osmolality (Advanced Instruments 3320 freezing-point osmometer), chloride (Haake Buchler digital chloridometer), and sodium and potassium (Cole-Palmer, model 410 single-channel flame photometer), as described by Farrell *et al.* (2001).

### Statistical analyses

Two-way analysis of variance (ANOVA) was used to test for differences in size (FL and *M*_B_) among treatment groups. No significant differences were found in either FL or *M*_B_ across groups (ANOVA: *P*>0.05), and so groups are compared directly herein. The effects of temperature and capture-and-release treatments on physiological variables were examined using two-way ANOVA. One fish in the 21°C and assisted ventilation group died within 20 min of blood sampling, 17 h ahead of all other mortality, and exhibited anomalous blood physiological variables. This individual was subsequently removed as an outlier in all analyses of physiological variables. Plasma cortisol values were analysed separately for sex in consideration of the naturally higher plasma cortisol values of migrating female sockeye salmon in comparison to migrating male sockeye salmon, as reported in the literature (Sandblom *et al.*, 2009; Hruska *et al.*, 2010; Roscoe *et al.*, 2011). Data for sodium, potassium, and haematocrit were log_10_ transformed to meet parametric assumptions. We used a power transformation on lactate values and a rank transformation on osmolality values in order to meet parametric assumptions. Significance levels were set at 0.05. Where significant differences were detected among simulated capture-and-release treatments, Bonferroni multiple comparisons tests were used. We focused our survival analyses at 10 and 15 days after the simulated capture-and-release treatment because these durations reflect a range of times it would take Early Summer and Summer-run sockeye salmon to reach their natal tributaries from our capture locale (Gilhousen, 1990). The percentage survival at 10 or 15 days was compared between simulated capture-and-release treatments of males and females at 16°C with Fisher's exact tests using Bonferroni corrections (*P* = 0.017).

## Results

The fisheries capture-and-release simulation significantly increased lactate, cortisol (male and female), glucose, osmolality, and haematocrit relative to control fish (Table 1). Female cortisol concentrations were roughly 2-fold greater than male concentrations of corresponding groups. Plasma potassium and MCHC significantly decreased in response to the capture-and-release simulation relative to control fish. The assisted ventilation technique did not have a significant effect on any blood variables when compared with the unassisted group, with the exception of plasma lactate at 21°C and haematocrit. In both instances, the magnitude of change, in reference to the control group, increased in the assisted ventilation group. The temperature treatment had a significant effect on plasma potassium, haematocrit, and haemoglobin. Treatment × temperature interactions were detected for plasma lactate and cortisol (males only; see Table 1).

**Table 1:** Sample sizes (*n*) and mean values ± SEM for physiological variables of sockeye salmon 30 min after one of three simulated fisheries capture-and-release treatments [control (C), no assisted ventilation (N), and assisted ventilation (A)] and two temperatures (16 and 21°C)

Physiological variable	Fisheries capture treatment	16°C		21°C		Treatment		Temperature		Interaction	
		*n*	Mean ± SEM	*n*	Mean ± SEM	*F*	*P*-value	*F*	*P*-value	*F*	*P*-value
Plasma lactate (mmol l^−1^)	C	18	1.65 ± 0.15^a^	15	2.62 ± 0.38^c^	250.1	<0.0001	0.9	0.3444	4.19	0.018
	N	18	13.38 ± 0.64^b^	16	11.73 ± 0.98^d^						
	A	19	13.06 ± 0.78^bd^	16	14.11 ± 0.98^b^						
Plasma cortisol (ng ml^−1^) in males	C	10	120.0 ± 19.6^a^	10	162.8 ± 29.3^a^	28.25	<0.0001	4.79	0.0335	4.71	0.0136
	N	10	387.5 ± 35.9^b^	8	268.3 ± 15.3^c^						
	A	10	350.5 ± 26.7^b^	6	273.6 ± 34.4^c^						
Plasma cortisol (ng ml^−1^) in females	C	8	301.0 ± 41.3^x^	5	339.8 ± 64.0	13.84	<0.0001	0.07	0.7885	0.64	0.533
	N	8	531.0 ± 59.7^y^	8	526.2 ± 57.1						
	A	9	586.1 ± 32.5^y^	10	521.5 ± 24.1						
Plasma glucose (mmol l^−1^)	C	18	6.10 ± 0.34^x^	15	7.06 ± 0.63	19.42	<0.0001	0.72	0.3988	0.51	0.6023
	N	18	9.33 ± 0.57^y^	16	9.15 ± 0.70						
	A	19	9.70 ± 0.59^y^	16	10.08 ± 0.47						
Plasma Na^+^ (mmol l^−1^)	C	18	155 ± 3	15	152 ± 5	1.22	0.2986	3.84	0.0531	0.27	0.7673
	N	18	159 ± 3	16	154 ± 3						
	A	19	163 ± 3	16	154 ± 3						
Plasma Cl^−^(mmol l^−1^)	C	18	131.3 ± 1.3	15	131.3 ± 1.8	2.07	0.1314	0.07	0.794	0.03	0.97
	N	18	134.1 ± 1.3	16	133.3 ± 2.3						
	A	19	134.9 ± 2.1	16	134.5 ± 1.0						
Plasma K^+^ (mmol l^−1^)	C	18	1.1 ± 0.1^x^	15	1.4 ± 0.2	17.36	<0.0001	11.77	0.0009	1.66	0.1955
	N	18	0.4 ± 0.04^y^	16	0.8 ± 0.1						
	A	19	0.6 ± 0.1^y^	16	0.8 ± 0.1						
Plasma osmolality (mosmol kg^−1^)	C	18	316 ± 2^x^	15	314 ± 3	33.7	<0.0001	0.15	0.6981	0.62	0.5388
	N	18	349 ± 3^y^	16	348 ± 3						
	A	19	343 ± 6^y^	16	350 ± 4						
Haematocrit (%)	C	18	41.1 ± 0.9^x^	15	41.7 ± 1.4	23.05	<0.0001	4.19	0.0434	1.35	0.2652
	N	18	44.6 ± 1.5^y^	16	49.9 ± 2.1						
	A	19	50.0 ± 1.0^z^	16	52.0 ± 1.6						
Haemoglobin (g l^−1^)	C	18	102 ± 2^x^	15	104 ± 3	4.56	0.0128	6.41	0.013	2.85	0.0628
	N	18	98 ± 3^xy^	16	112 ± 4						
	A	19	110 ± 2^y^	16	112 ± 3						
Mean corpuscular haemoglobin concentration (MCHC; g l^−1^)	C	18	249 ± 3^x^	15	252 ± 6	34.57	<0.0001	0.09	0.7641	0.74	0.4816
	N	18	221 ± 3^y^	16	226 ± 4						
	A	19	222 ± 4^y^	16	217 ± 4						

Two-way ANOVA results are presented for each physiological variable. The superscript letters present the results of Bonferroni multiple comparisons tests. Dissimilar lower-case letters denote significant differences between values, where a–d denote differences with respect to significant treatment × temperature interactions and x–z denote significant differences with respect to the treatment main effect across both temperatures.

**Figure 1:**
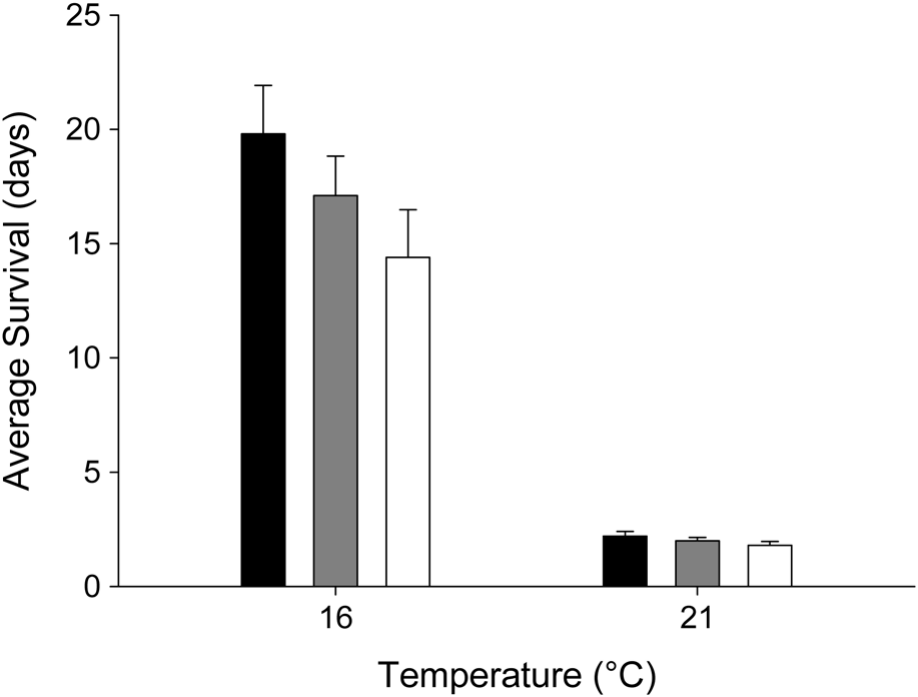
Average days survived after experimental treatment [control (filled bars), fisheries capture-and-release simulation without assisted ventilation (shaded bars), and fisheries capture-and-release simulation with assisted ventilation (open bars)] for sockeye salmon held at 16 and 21°C, with standard error bars (*n* = 15–19).

**Figure 2:**
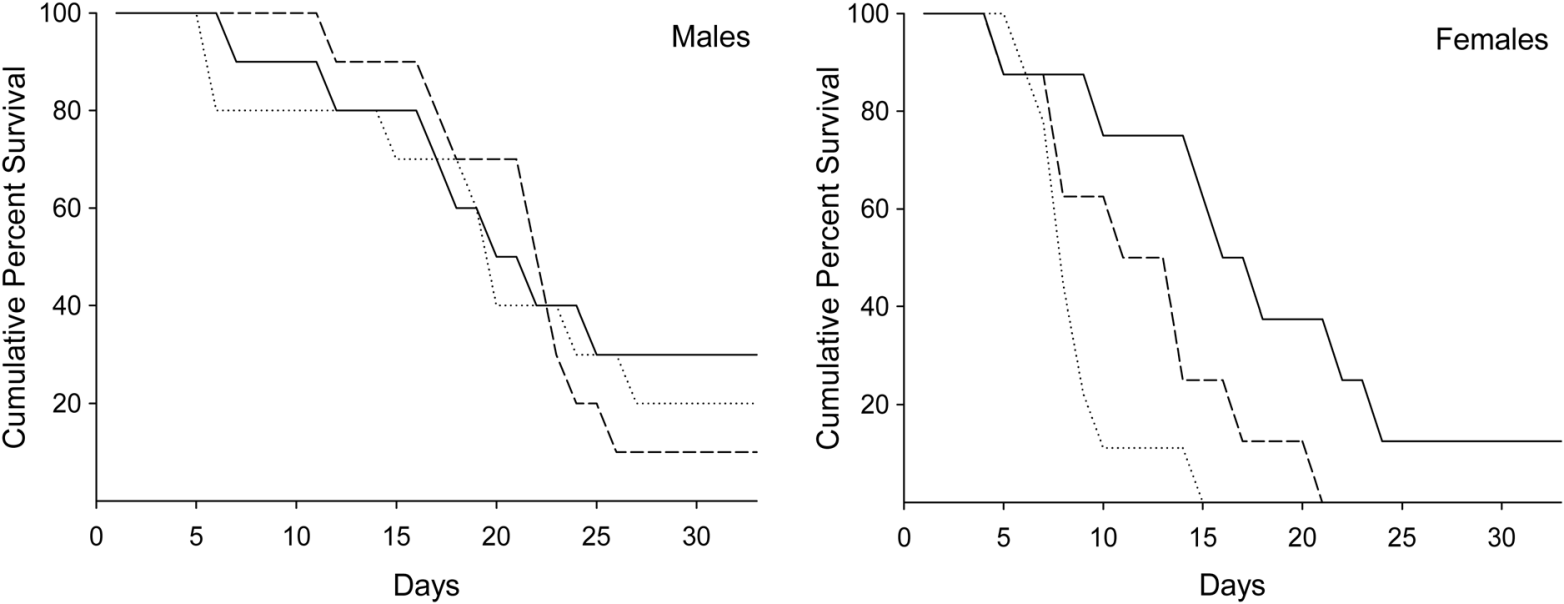
Cumulative percentage survival after experimental treatment for male and female sockeye salmon held at 16°C (*n* = 8–10). Seven fish (six males and one female) remained at the end-point of the experiment (33 days). The type of line indicates the simulated fisheries capture-and-release treatment [control (continuous line), fisheries capture-and-release simulation without assisted ventilation (dashed line), and fisheries capture-and-release simulation with assisted ventilation (dotted line)].

**Figure 3:**
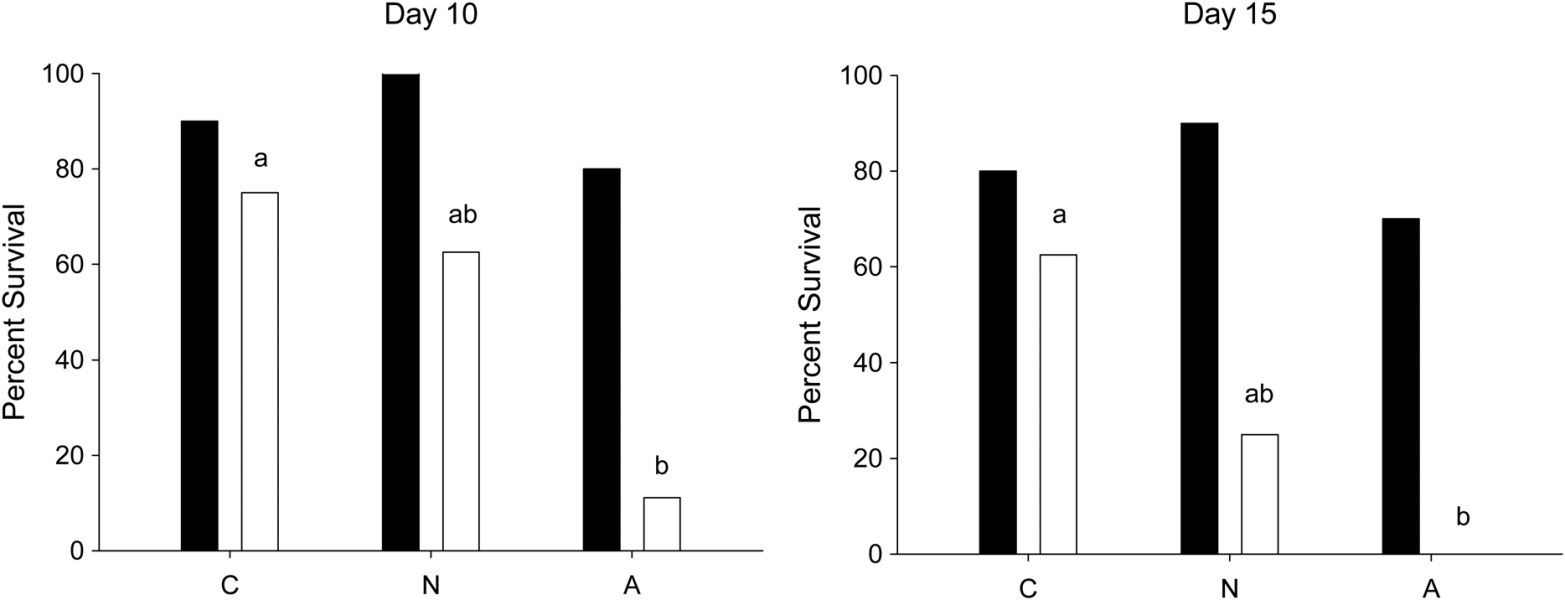
Percentage survival 10 and 15 days after experimental treatment [control (C), no assisted ventilation (N), and assisted ventilation (A)] for males (filled bars; *n* = 10) and females (open bars; *n* = 8–9) held at 16°C. Different lower-case letters indicate significant differences between simulated fisheries capture-and-release treatments after Bonferroni corrections for multiple comparisons. Significant differences were not detected among male treatment groups.

Fish held at 21°C exhibited 100% mortality across all groups within 3 days after the simulated capture-and-release treatments (Fig. 1). At 16°C, mortality began 4 days after the simulated capture-and-release treatments and continued until the end-point of the experiment (33 days after treatment), when seven fish remained. Females that were subjected to manual ventilation assistance exhibited poor survival in comparison with unassisted and control females, and compared with males of all treatments (Fig. 2). At day 10, 11% (one of nine) of females that received ventilation assistance survived compared with 63% (five of eight) of females that did not receive ventilation assistance and 75% (six of eight) of control females (Fig. 3). By day 15, females from both of the capture-and-release simulation groups exhibited poor survival [0% (zero of nine) for the assisted group and 25% (two of eight) for the unassisted group] compared with the female control fish [63% (five of eight); Fig. 3]. There were no significant differences in survival for males between treatment groups at either day 10 or day 15. By day 15, males across all groups exhibited 70–90% survival.

## Discussion

This study is one of the first assessments of the long-term survival benefits of holding fish facing into a water currentprior to release, despite the fact that this technique is routinely used by conservation-minded anglers and encouraged by many natural resource agencies (Pelletier *et al.*, 2007). The method of ventilation assistance used here did not enhance the survival of sockeye salmon after the simulated capture-and-release event. In the high-temperature group, which approximates peak summer water temperatures in the Fraser River in recent years, none of the fish survived beyond 3 days, regardless of the simulated capture-and-release treatments. At a temperature near the current summer average for the Fraser River, females exhibited an increase in mortality in response to the simulated fisheries capture and subsequent assisted ventilation technique.

Using manual ventilation assistance as a technique for facilitating recovery is based on the idea that forcing water over the gill surface is effective in aiding oxygen uptake at a time when excess oxygen is needed for recovery. The plasma variables measured here clearly indicate that the fisheries capture-and-release simulation was sufficient to cause metabolic disruption. Plasma cortisol, the principal corticosteroid measured in response to capture-and-release stressors, was significantly higher for fish that were exposed to the capture-and-release simulation compared with control fish. Moreover, the significant increase in glucose concentrations for these fish is consistent with the mobilization of energy stores triggered by stress hormones (e.g. cortisol; Gamperl *et al.*, 1994; Wendelaar Bonga, 1997). Most importantly, plasma lactate following the simulation was 6-fold greater than the control values (∼2 mmol l^−1^). The level reached with the capture-and-release simulation (∼13 mmol l^−1^ with or without assisted ventilation) exceeded that proposed to be characteristic of anaerobic activity during burst swimming in sockeye salmon (i.e. concentrations >6 mmol l^−1^; Eliason Parsons, 2011) and reached the level proposed as the threshold for negatively affecting repeat swim performance in rainbow trout (Jain and Farrell, 2003). This physiological evidence supports a reasonable expectation that post-capture ventilation assistance would be beneficial for fish recovering from the exhaustive exercise associated with capture-and-release events.

The incorporation of two ecologically relevant water temperatures into this study was done to assess whether the current warming trends observed in the Fraser River will affect attempts to facilitate recovery after capture. Sockeye salmon that were held at the record peak water temperature (21°C) exhibited rapid mortality with no significant differences among the capture-and-release treatments, including control fish. Studies have shown that water temperatures >18°C have negative survival consequences on sockeye salmon during their freshwater spawning migration (Naughton *et al.*, 2005; Keefer *et al.*, 2008; Martins *et al.*, 2011). Several mechanisms responsible for this mortality have been proposed, including the depletion of energy stores (Rand *et al.*, 2006), the increased progression of pathogens and disease (Crossin *et al.*, 2008; Bradford *et al.*, 2010), and the collapse of aerobic scope and cardiovascular function (Farrell *et al.*, 2008). Regardless of the exact cause, the present results emphasize the deleterious effects of warming migration temperatures on Fraser River sockeye salmon and are particularly relevant as Fraser River water temperatures are expected to continue to rise (Morrison *et al.*, 2002).

At the more moderate temperature of 16°C, the assisted ventilation technique was not effective in enhancing survival after our capture-and-release simulation. Moreover, the females that were exposed to the fisheries simulation with manual ventilation assistance exhibited the highest mortality rate among all treatment groups. This relatively high mortality suggests that the females were particularly vulnerable to the additional stress of handling in our experiment. Migrating female sockeye salmon have higher plasma cortisol values in comparison to males (Sandblom *et al.*, 2009; Hruska *et al.*, 2010; Roscoe *et al.*, 2011), largely due to the role of cortisol in reproductive maturation and assisting in the mobilization of energy stores for gonad development (Mommsen *et al.*, 1999). A potential consequence of this cortisol elevation is an increased susceptibility to infection (Martins *et al.* 2012) because plasma cortisol also acts as an immunosuppressant (Schreck *et al.*, 2001). The increased mortality of female sockeye salmon relative to males is consistent with other studies on migrating Fraser River sockeye salmon (Patterson *et al.*, 2004; Crossin *et al.*, 2008; Nadeau *et al.*, 2010; Roscoe *et al.*, 2011; Jeffries *et al.*, 2012; Martins *et al.*, 2012).

Previous studies evaluating methods of enhancing post-release survival of Pacific salmon using recovery devices suggest that any benefit of using these devices must be balanced with the potential for additional stress resulting from added handling and confinement (Farrell *et al.*, 2001; Donaldson *et al.*, 2013; Nguyen *et al.*, 2013). Thus, the attempt to restrain fish manually for 1 min may have compounded the physiological stress that this approach was attempting to alleviate, particularly as elevated cortisol will prolong the removal of lactate and the resynthesis of glycogen after anaerobic burst activity (Pagnotta *et al.*, 1994; Eros and Milligan, 1996; Milligan *et al.*, 2000). However, considering the single sampling point in this experiment and the dynamic process of the cortisol response trajectory (Barton, 2002), it cannot be determined whether the extra handling required for this method of ventilation assistance resulted in a prolonged elevation of cortisol concentrations and thus a delay in recovery.

The present laboratory study removed some of the natural variables associated with sockeye salmon migration through the Fraser River after release from capture (e.g. hydraulic challenges, secondary fisheries capture, and predation) and enabled long-term monitoring, which led to the discovery of a sexual dichotomy in post-release survival. However, holding fish in tanks results in confinement stress not experienced in natural conditions (Roscoe *et al.*, 2011) and this confinement may be particularly harmful for female sockeye salmon (Sandblom *et al.*, 2009; Jeffries *et al.*, 2012). Laboratory studies will continue to offer a great deal of control over experimental treatments and environmental conditions (Cooke *et al.*, 2013); however, combining these results with long-term biotelemetry monitoring of wild fish after release will allow for a more complete evaluation of capture-and-release events and assisted ventilation techniques (Donaldson *et al.*, 2008, 2013).

In summary, most North American recreational fisheries regulations recommend a method of manually reviving fish intended to be released after capture (Pelletier *et al.*, 2007). The manual ventilation assistance that was examined here was designed to simulate the attempts at recovery that would be implemented by Fraser River recreational anglers. The lack of any benefit and the potential for female-specific mortality after using the ventilation assistance described herein provides an example of the need to test assumptions regarding universal best practices for handling sockeye salmon in moderate temperatures. Furthermore, this study reiterates that high water temperature can have a profound influence on the survival of fish, particularly when associated with handling. Managers can now evaluate the fisheries implications of matching the future climate scenarios that indicate an increase in frequency and duration of high water temperatures for the Fraser River (Hague *et al.*, 2011) with the temperature-related mortality that overshadows any attempt at facilitating recovery after capture.
